# In the View of Endothelial Microparticles: Novel Perspectives for Diagnostic and Pharmacological Management of Cardiovascular Risk during Diabetes Distress

**DOI:** 10.1155/2018/9685205

**Published:** 2018-05-13

**Authors:** Larissa Pernomian, Jôsimar Dornelas Moreira, Mayara Santos Gomes

**Affiliations:** ^1^Department of Biosciences Applied to Pharmacy, Faculty of Pharmaceutical Sciences from Ribeirão Preto, University of São Paulo, Ribeirão Preto, SP, Brazil; ^2^Department of Clinical and Toxicological Analysis, Faculty of Pharmacy, Federal University of Minas Gerais, Belo Horizonte, MG, Brazil; ^3^Department of Physics and Chemistry, Faculty of Pharmaceutical Sciences from Ribeirão Preto, University of São Paulo, Ribeirão Preto, SP, Brazil

## Abstract

Acute or chronic exposure to diabetes-related stressors triggers a specific psychological and behavior stress syndrome called diabetes distress, which underlies depressive symptoms in most diabetic patients. Distressed and/or depressive diabetic adults exhibit higher rates of cardiovascular mortality and morbidity, which have been correlated to macrovascular complications evoked by diabetic behavior stress. Recent experimental findings clearly point out that oxidative stress accounts for the vascular dysfunction initiated by the exposure to life stressors in diabetic conditions. Moreover, oxidative stress has been described as the main autocrine and paracrine mechanism of cardiovascular damage induced by endothelial microparticles (anuclear ectosomal microvesicles released from injured endothelial cells) in diabetic subjects. Such robust relationship between oxidative stress and cardiovascular diseases strongly suggests a critical role for endothelial microparticles as the primer messengers of the redox-dependent vascular dysfunction underlying diabetes distress. Here, we provide novel perspectives opened in the view of endothelial microparticles as promising diagnostic and pharmacotherapeutic biomarkers of cardiovascular risk in distressed diabetic patients.

## 1. Introduction

Diabetes distress has been described as a psychological and behavior stress syndrome of emotional symptoms that affect almost 50% of total diabetic patients [1–7]. Distressed diabetic patients exhibit higher rates of cardiovascular mortality and morbidity, which suggest a strong positive correlation between diabetes distress and cardiovascular risk of developing hypertension, dyslipidemia, atherosclerosis, myocardial infarction, stroke, and sudden death [8–15]. A few recent studies have shown that oxidative stress seems to be the key regulator of diabetes distress-induced cardiovascular diseases by impairing local anti-inflammatory nitrergic signaling [3, 16–20]. Beyond the redox-mediated cardiovascular dysfunction proposed in diabetes distress, submicron vesicles shed from activated or quiescent endothelial cells, called endothelial microparticles (EMPs), have been pointed out as the main mediators of cardiovascular dysfunction underlying isolated diabetes by inducing proinflammatory pathways. In brief, EMP generation is increased by the typical oxidative stress induced in diabetic subjects affected by diabetes-related cardiovascular complications [21–25]. Taken together, these findings suggest that EMPs could represent a putative link between diabetes distress and the underlying redox-mediated cardiovascular dysfunction. Since there are no reports regarding the potential role assigned to EMPs as the downstream mediators of diabetes distress-related cardiovascular diseases, here we provide hypothetical mechanistic insights thereon by opening novel perspectives for the forthcoming use of EMPs as a diagnostic tool and pharmacological target for managing cardiovascular risk in distressed diabetic patients.

## 2. Diabetes Distress: Can Stress Hurt?

Considering that this question is not as simple as it seems to be, the more appropriate answer would be: it depends on how long stress lasts and how regulated the stress-based reactions are. If we understand psychological stress as an adaptive behavior resultant from transient neurological and hormonal responses induced for the sole purpose of ensuring survival [26], we would conclude that stress protects instead of hurts. However, if stress lasts for a long enough time and/or its reactions run on unregulated pathways, it can certainly hurt by triggering or contributing to the advance of a pathological environment [27]. In both cases, the outcomes of psychological stress arise from the hyperactivation of the hypothalamic-pituitary axis (HPA), whose tissue and systemic effects comprise extensive transcriptional and epigenetic changes [28].

In the view of diabetes, there are innumerous disease-related stressors that acutely or chronically activate HPA in an unregulated fashion, which leads to irregular peaks of serum corticosterone [29]. Overall, diabetes-related stressors comprise the following: significant emotional reactions to the diagnosis; requirement of self-management; blame and denial in dealing with the disease, the oppressive self-management, and the social restrictions surrounding diabetic conditions; threats of diabetic complications; general diabetic depletion; and potential loss of function [1–3]. The irregular corticosterone peaks induced by diabetes-related stressors feedback their own behavior effects [30], leading to an expected stress reaction called diabetes distress, which rises as an affective experience involving wide emotional responses such as fears, worries, concerns, blame, and burden [2–7]. In addition to the behavior effects, the irregular corticosterone peaks induced by diabetes-related stressors also enhance diabetic insulin resistance and hyperglycemia, which contribute to the progression of diabetes [30]. For these reasons, diabetes distress is also correlated to a suboptimal glycemic control [4, 31, 32].

Diabetes distress is a very common condition so that up to 45% of type 2-diabetic patients experience its emotional symptoms [1–3]. This high incidence reduces the complacence of distressed diabetic patients for pharmacotherapy, diet, or physical exercises in 5–15%, 30%, or 80%, respectively [33]. For these reasons, these subjects exhibit higher rates of mortality correlated to all causes [34].

Although diabetes distress is a specific psychological and behavior stress syndrome that belongs to the diabetes spectrum, it has been described as a nonspecific indicator of clinical psychiatric comorbidities since it underlies major depression, elevated depressive symptoms, and subclinical depression that are widely developed by diabetic patients [2–7]. About 20–30% of distressed diabetic patients experience clinical depressive symptoms [4, 35] so that the prevalence of major depression is two- or three-fold higher in type 2- or type 1-diabetic patients than in the general population, respectively [3, 36]. Both diabetes distress and major depression prognosis worsen when the two conditions coexist [37]: while HPA hyperactivity in distressed diabetic patients contributes to the progression of clinical depression, sympathomedular activation in depressive patients contributes to the insulin resistance during diabetes progression [38]. Taken together, these considerations support the importance of appropriately treating distressed diabetic patients in an attempt to avoid stress hurting. For these purposes, an effective glycemic control associated to behavior-cognitive approaches for improving diabetic prognosis and controlling the emotional stress and the eventual coexistent psychiatric symptoms has been considered one of the most effective choices [39].

## 3. Diabetes Distress and Cardiovascular Risk: What Is in Common?

There is much in common between diabetes distress and cardiovascular risk, starting with their prevalence as comorbidities. It is well documented that cardiovascular diseases represent the major cause of morbidity and mortality among diabetic patients, who exhibit a two-fold higher cardiovascular risk than the normoglycemic population [16, 40]. However, this higher incidence does not depend only on the traditional risk factors for cardiovascular diseases but it is strongly correlated to the behavior risk factors [40], pointing at diabetes distress as the major cause of cardiovascular complications underlying chronic hyperglycemia in stressed diabetic subjects [8].

Accordingly, distressed diabetic patients have a 1.69-fold higher risk for developing cardiovascular events [9], a 1.8-fold higher risk for developing cardiac ischemic diseases [10], and a 1.76-fold higher risk of cardiovascular mortality [9]. When diabetes distress is followed by clinical psychiatric disorders, cardiovascular risk gets a substantial increase of 39% [11] so that distressed depressive diabetic patients have a 2.4- to 3.5-fold higher risk of cardiovascular mortality [12]. Hypertension and macrovascular complications (myocardial infarction, stroke, and sudden death) are the most expressive cardiovascular events expressed in these patients, counting on a risk of 56% [13] and 24–82% [14, 15], respectively.

A marked positive correlation between diabetes distress and the main biomarkers of cardiovascular diseases (i.e., glycosylated hemoglobin (HbA1c), diastolic pressure, and low-density lipoprotein-cholesterol (LDL-c)) was recently confirmed in the most common cardiovascular events expressed in these patients [3, 16]. Accordingly, several studies have attempted to elucidate the mechanisms underlying the increased cardiovascular risk in diabetes distress. In this sense, recent studies have confirmed the critical role played by oxidative stress in exacerbating vascular diseases during experimental diabetes distress, which is induced by the behavior restraint stress model. Moreira et al. [17, 18] have showed that the contractile carotid hyperreactivity underlying cerebrovascular complication in stressed diabetic animals involves the upregulation of NADPH/Nox4 oxidase-driven generation of hydrogen peroxide (H_2_O_2_) [17] and inducible nitric oxide synthase (*i*NOS)-driven generation of peroxynitrite (ONOO^−^) [18]. The authors have demonstrated that the increased generation of H_2_O_2_ and ONOO^−^ is induced by the acute exposure of rats to restraint stress, which increases carotid resistance by enhancing the local preexistent diabetes-related angiotensinergic hyperreactivity [19] due to the contractile effects evoked by the referred reactive oxygen species (ROS) [17, 18]. In agreement with these findings, Matsuura et al. [20] showed that the chronic exposure of animals with metabolic syndrome (DahlS.Z-Lepr^fa^/Lepr^fa^/obese rats) to restraint stress exacerbates the preexisting hypertension, ventricular hypertrophy fibrosis, and diastolic dysfunction by cardiovascular oxidative stress- and proinflammatory-dependent mechanisms and hyperactivation of the local renin-angiotensin system (RAS). The clinical and experimental findings regarding the biomarkers of diabetes distress-related cardiovascular risk are summarized in Table 1.

The experimental correlations assigned to renin-angiotensin system (RAS) activation and redox signaling suggest vascular dysfunction as the putative key mechanism underlying the increased cardiovascular risk during diabetes distress [3, 16, 17, 20]. Indeed, angiotensinergic oxidative stress has been described as a critical mechanism for inducing general vascular dysfunction under both stressful [41–44] and hyperglycemic [19] stimuli. In turn, vascular dysfunction underlies all of the diabetic cardiovascular complications during their early preclinical stages [45]. Thus, the identification of integrative molecular players of redox-dependent vascular dysfunction provides a novel pathophysiological understanding of cardiovascular risk in diabetes distress and novel insights for selectively targeting vascular competence for diagnosis, prognosis, and therapy purposes.

## 4. Cardiovascular Risk in Diabetes Distress and Endothelial Microparticles: Could They Be Linked?

When taking oxidative stress into account, cardiovascular risk in diabetes distress can certainly be linked to endothelial microparticles (EMPs), which have been described as noninvasive surrogate biomarkers of redox-induced vascular injury and dysfunction correlated with poor clinical outcomes in cardiovascular diseases [45]. In brief, EMPs are submicron vesicles ranging from 0.1 to 1.0 *μ*m in diameter, shed from the blebbing of plasma membrane of activated or quiescent endothelial cells during prooxidant events that lead to a direct effect of ROS on the vasculature such as shear stress-induced blood pressure disorders (which induces ROS generation upon NADPH oxidase activation), the agonist activation of receptors attached to redox signaling (such as angiotensin AT_1_, endothelin ET_A_, endothelin ET_B_, and TNF*α*-I receptors), the redox-induced cell apoptosis, and/or the oxidative stress-mediated mitochondrial dysfunction [21, 46–48]. As autocrine and paracrine mediators, EMPs elicit oxidative stress in endothelial cells by increasing the local ROS generation and triggering underlying redox-proinflammatory mechanisms that (1) downregulate nitric oxide (NO) release by inducing the uncoupling of endothelial nitric oxide synthase (*e*NOS) and (2) disrupt the endothelium competence on nitrergic anti-inflammatory signaling [49]. This effect may be due to the expression of functionally active NADPH oxidase subunits (*p*22^phox^, *p*38^phox^, *p*47^phox^, *p*67^phox^, Nox1, Nox2, and Nox4) in EMPs [50].

**Figure 1:**
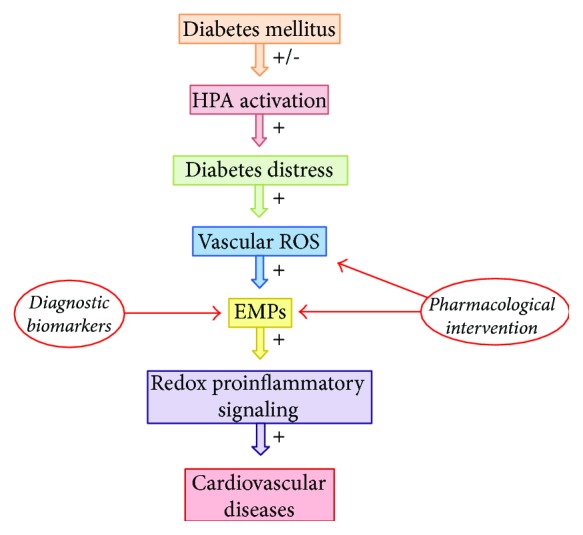
Hypothetical mechanistic insights regarding the potential role played by endothelial microparticles as key downstream mediators of diabetes distress-related cardiovascular diseases. The irregular activation of HPA in diabetic patients leads to the psychological and behavior stress syndrome called diabetes distress, which induces vascular oxidative stress by enhancing the functionality of the renin-angiotensin system and NADPH oxidase; the resultant ROS activate EMP generation upon redox-mediated quiescence of endothelial cells; finally, EMPs trigger redox proinflammatory signaling in autocrine and paracrine mechanisms that underlies cardiovascular diseases positively correlated with diabetes distress. Such putative role assigned to EMPs opens novel perspectives in using the generation of EMPs as a biomarker and a target for diagnostic and pharmacological intervention aimed at the managing of diabetes distress-related cardiovascular risk, respectively. HPA (hypothalamic-pituitary axis); ROS (reactive oxygen species); EMPs (endothelial microparticles).

Circulating EMPs represent the minority of total microparticles in healthy conditions but their levels are substantially increased by the oxidative stress-induced lipid peroxidation in endothelial cells during diabetes or cardiovascular diseases [21–25]. Moreover, diabetic cardiovascular complications increase the circulating levels of EMPs when compared to isolated diabetes or cardiovascular diseases [51, 52]. Interestingly, diabetes seems to increase EMPs' competence in inducing vascular dysfunction. Jansen et al. [53] recently showed that the redox-dependent vascular inflammation in atherosclerotic lesions (i.e., macrophage infiltration and adhesion protein expression) is higher when induced by EMPs derived from endothelial cells exposed to diabetes-like hyperglycemic conditions. Accordingly, although high glucose levels did not change EMPs' morphology (i.e., size and antigen composition), the hyperglycemia increases the functionality of EMPs in activating NADPH oxidase/*p*38^phox^-mediated signaling when compared to EMPs formed in a normoglycemic environment [53]. The clinical significance of such redox/proinflammatory potential assigned to diabetic EMPs lies in their prognostic value for coronary artery disease in diabetes due to the positive correlation between the high circulating EMP levels and the severity of vascular dysfunction in diabetic patients [23, 54, 55]. Taken together with the well-described remarkable increase in vascular inflammation, vascular ROS generation, and vascular NAPH oxidase complex expression and functionality in animal models of diabetes distress [17–20], the stronger competence of diabetic EMPs in inducing oxidative vascular injury and dysfunction points at EMPs as feasible integrative molecular players of the increased cardiovascular risk in diabetes distress [8–16, 40]. Such hypothetical mechanistic insights are summarized in Figure 1. Therefore, selectively targeting EMPs may represent a promising perspective for effective diagnostic, prognostic, and therapeutic purposes aimed at the cardiovascular risk in distressed diabetic patients.

## 5. In the view of Endothelial Microparticles: Novel Insights for Cardiovascular Risk Management in Diabetes Distress!

The recent findings described here concerning the oxidative potential assigned to EMPs in inducing diabetic cardiovascular complications open real perspectives for managing cardiovascular risk during diabetes distress by considering EMPs as diagnostic key hallmarks and pharmacological targets. For diagnostic purposes, the circulating levels of EMPs can be quantified by polychromatic flow cytometry as the simplest method that provides valuable information regarding the antigen composition and size criteria [56, 57]. In these cases, platelet-poor plasma must be obtained by serial centrifugation or ultracentrifugation of freshly drawn whole blood samples, since freezing or storage approaches disrupt the microparticles' integrity. Also, the blood samples must be pretreated with calcium (Ca^2+^)-chelator anticoagulants (e.g., EDTA or sodium citrate) for avoiding microparticle aggregation. Once prepared, the platelet-poor plasma is labeled with fluorescent monoclonal antibodies for staining specific surface antigens expressed on endothelial cells such as CD31 and CD54, and the cytometric analysis is performed by applying forward and side light scatter with calibration beads of a known diameter [58].

A few recent studies have shown that some specific pharmacological interventions are able to promote protective effects by significantly reducing circulating levels of EMPs in diabetic patients affected by cardiovascular diseases including hypertension. For instance, the Ca^2+^ blockers nifedipine and benifipine, combined or not with the angiotensin AT_1_ antagonist losartan, were able to reduce EMP formation in hypertensive diabetic patients by antioxidant mechanisms that inhibited lipid peroxidation in endothelial cells rather than hypotensive effects [59–61]. Similarly, statins such as simvastatin and fluvastatin, as well as vitamin C and losartan, reduced the circulating levels of EMPs in hypertensive hyperlipidemic diabetic patients affected by advanced atherosclerotic plaques [22, 60, 62–64]. Interestingly, the therapeutic effects of these drugs on EMP circulating levels result from pharmacological actions that interfere with the mechanisms of EMP formation. Indeed, it would not be feasible to selectively target EMPs with specific antibodies or scavengers since other microparticles stemmed from different parental cells share some of the surface antigens expressed by EMPs including CD31 and CD54 [65]. Currently, these mechanisms are summarized as the shedding of activated or quiescent endothelial cell plasma membrane blebbing, which can result from the increase of intracellular Ca^2+^ or ROS levels induced by shear stress, agonist receptor activation (including the activation of angiotensin AT_1_, endothelin ET_A_, endothelin ET_B_, and TNF*α*-I receptors), apoptosis, or oxidative stress-mediated mitochondrial dysfunction [21, 46–48, 66–68]. However, recent studies have shown that lipid-lowering therapies inhibit EMP formation by an unknown mechanism [69, 70] that putatively interferes with the Rho-kinase pathway [63]. Such information highlights the importance of accurately identifying all the pathophysiological mechanisms of EMP formation so that they can be defined as pharmacological targets for controlling cardiovascular risk in distressed diabetic patients.

Although there are no reports concerning the direct effects of selective serotonin reuptake inhibitors (IRS) on circulating EMP levels from depressive distressed diabetic patients [71], experimental findings have shown that escitalopram and paroxetine prevent vascular dysfunction in chronically stressed or diabetic rats by cardiac and endothelial antioxidant mechanisms [72–74]. Taken together, these findings suggest a novel therapeutic role for IRS as protective agents on the cardiovascular system by putatively inhibiting the redox-dependent formation of EMPs during stressful and/or diabetic conditions. The confirmation of this hypothesis would then make IRS an important pharmacological tool as reliable as Ca^2+^ blockers, statins, AT_1_ antagonists, or antioxidants for managing cardiovascular risk in diabetes distress.

## Figures and Tables

**Table 1:** Biomarkers of diabetes distress-related cardiovascular risk.

Study	Biomarker	Cardiovascular disease	References
DD-T2DM patients	↑ HbA1c	Microvascular outcomes	Kreider [3]
	↑ Diastolic pressure	Macrovascular outcomes	Winchester et al. [16]
	↑ LDL-c	Hypertension	
		Dyslipidemia	
		Atherosclerosis	

AS-T1DM rats	↑ Vascular NADPH oxidase/Nox4 functionality	Carotid disease	Moreira et al. [17, 18]
	↑ Vascular Nox4-derived H_2_O_2_ levels	Stroke	
	↑ Vascular iNOS functionality		
	↑ Vascular iNOS-derived ONOO^−^ levels		
	↑ Vascular RAS functionality		

CS-DS/obese rats	↑ Cardiac NADPH oxidase functionality		Matsuura et al. [20]
	↑ Cardiac NADPH oxidase-derived O_2_^−^ levels		
	↑ Cardiac RAS functionality		
	↑ Cardiac macrophage infiltration		
	↑ Cardiac inflammation		

DD-T2DM patients (distressed depressive patients with type-2 diabetes mellitus); AS-T1DM rats (acutely stressed rats with type 1 diabetes mellitus); CS-DS/obese rats (chronically stressed rats with metabolic syndrome); HbA1C (glycosylated hemoglobin); LDL-c (low-density lipoprotein-cholesterol); H_2_O_2_ (hydrogen peroxide); iNOS (inducible nitric oxide synthase); ONOO^−^ (peroxynitrite); RAS (renin-angiotensin system); O_2_^−^ (superoxide anion).
